# Multimodal electrospray thruster for small spacecraft: design and experimental characterization

**DOI:** 10.1007/s44205-024-00075-0

**Published:** 2024-07-01

**Authors:** Peter Mallalieu, Manish Jugroot

**Affiliations:** RMC Advanced Propulsion and Plasma Exploration Laboratory (RAPPEL), Department of Mechanical and Aerospace Engineering, Royal Military College of Canada, 13 General Crerar Crescent, Kingston, K7K 7B4 Ontario Canada

**Keywords:** Electric propulsion, Multimodal thruster, Spacecraft propulsion, Electrospray propulsion, Ionic liquids, Ion-mode propulsion, Small spacecraft

## Abstract

Electrospray thrusters are a promising electric micropropulsion technology which could be used to meet the propulsion needs of nanosatellites, or for fine attitude control of larger spacecraft. Multimodal propulsion is the integration of two or more propulsion modes into a system which utilizes a common propellant. Indeed, spacecraft mission simulations and models have shown that this type of multimode propulsion capacity is exciting because of the flexibility and adaptability it provides mission designers and planners. A single spacecraft would have potential to execute drastically different mission profiles, and the exact mission could even be determined post-launch. The current paper investigates a micro-propulsion system which combines a droplet and ion mode electrospray emitter into a unified multimodal system (using an ionic liquid as the common propellant for both systems). The high relative thrust droplet mode emitter was fabricated from P3 borosilicate glass while the high efficiency ion mode emitter, Carbon Xerogel dense porous substrate, was fabricated in-house. To characterize the multimodal thruster, a full beam and time-of-flight (ToF) experimental setup were developed at the RMC Advanced Propulsion and Plasma Exploration Laboratory (RAPPEL) and experiments were conducted using a custom vacuum chamber. The ion mode emitter, with a beam comprised purely of ions had an onset voltage around 1400 V with an estimated thrust performance of 0.14 $\mu N$ and specific impulse of 4040 s. For droplet mode, with a mixed beam comprised of around 17$\%$ droplets and 83$\%$ ions, an onset voltage of 1375 V with an estimated performance of thrust at 14 $\mu N$ and specific impulse of 140 s were measured. The prototype thruster demonstrates how various electrospray emitters could be combined into a multimodal system to provide flexibility and adaptability in providing effective thrust for small satellites.

## Introduction on electrosprays and multimodal propulsion

Recently, there has been a trend towards launching small satellites into space. One common example is the CubeSat concept introduced to create a common standard form for small satellites with the goal of being compact, lightweight, and using standard commercial off-the-shelf (COTS) components [1]. While satellites have scaled down in size, it has been a challenge to develop propulsion systems which are capable of operating in a low power regime with a small form factor. The scaling of a thruster’s physical size does not lead to a linear change in performance - this challenge has led to the vast majority of operational small satellites having no propulsion capability [2].

Electrospray thrusters are an emerging form of electric propulsion (EP), with the ability of producing thrust for precision attitude control on larger satellites [3], and potentially for a wider array of orbital maneuvers on small satellites [4]. Electrosprays use a strong electric field formed by applying a large voltage difference between an emitter and an extraction grid to eject electrically conductive propellants producing thrust. The electric field deforms the propellant into a liquid meniscus, extending outward towards the extractor grid in a cone shape from which particles are ejected [5]. Electrosprays have several benefits compared to other forms of EP, as there is no requirement for an ionization process. Furthermore, the propellant can be fed from a reservoir to the emitters by capillary action, therby eliminating the need for pressurized components or pumps. Thirdly, since propellants such as ionic liquids (IL) contain both anions and cations, electrospray thrusters have the ability to produce a quasi-neutral beam, which therefoe allows operation without a neutralizer component (necessary to many other forms of EP).

Electrospray thrusters have several different modes of operation as investigated in the current paper. When purely emitting liquid droplets, the thruster considered to be operating in droplet mode. On the other hand, when an electrospray thruster emits purely ions, it is said to be operating in an ion mode or in the purely ionic regime (PIR). IL propellants are commonly used in ion mode electrosprays due to their excellent vacuum properties and ability to produce ion emission [6]. The thrust and specific impulse of a thruster depend upon the mode of operation: droplet mode produces a higher relative thrust, while ion mode produces a higher specific impulse in the range of several thousand seconds.

**Fig. 1:**
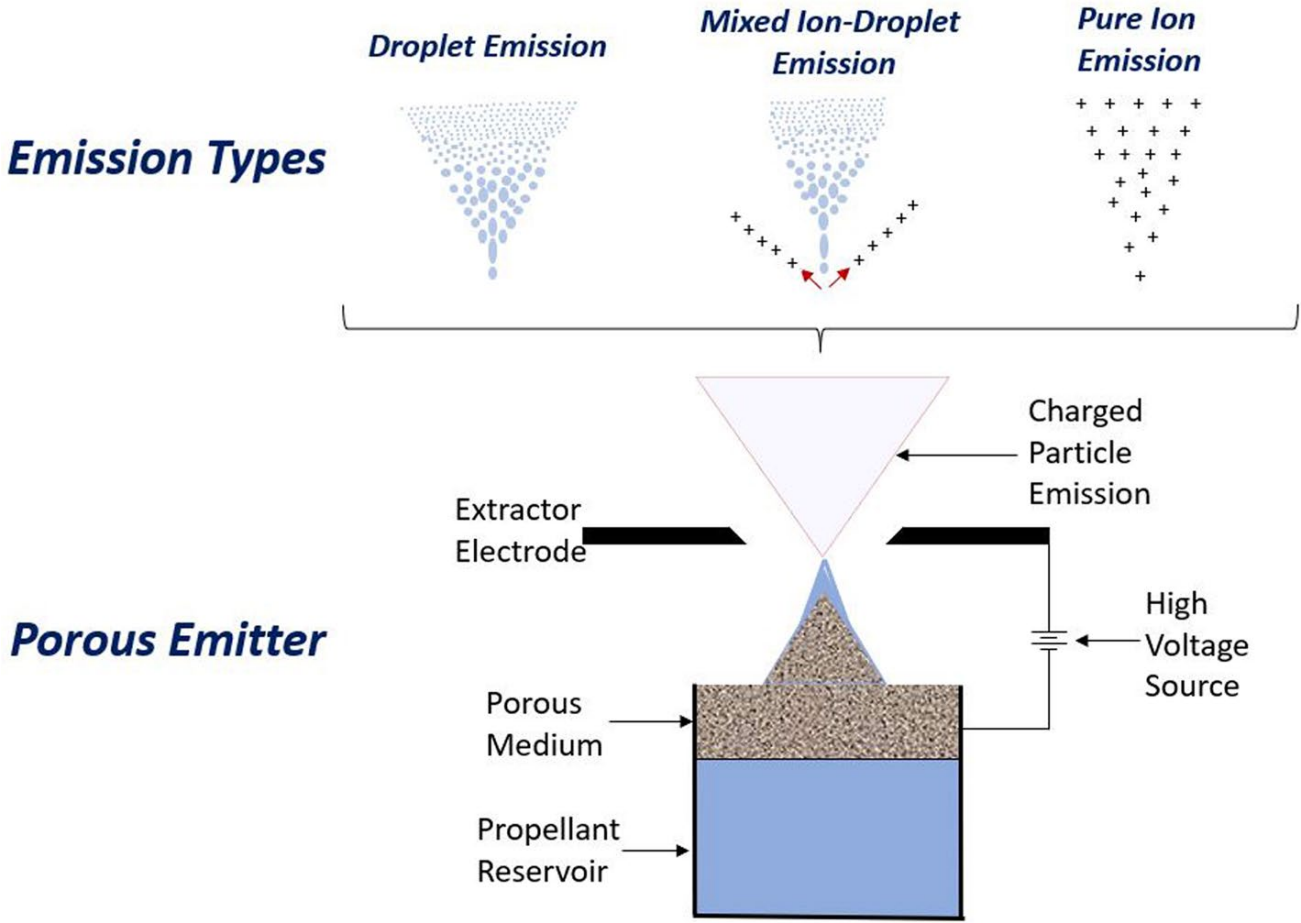
The three common types of electrospray emitters used for propulsion

Porous electrospray emitters are currently used in state-of-the-art designs focusing on ion mode emission [7, 8]. Since the liquid travels between the pores of the material, it is not directly exposed to its surroundings. A material’s porosity and mean pore size influence the attainable propellant flow rate. When using porous emitters, liquid propellant is most often transported from the reservoir to the emission site passively through capillary action. Figure 1 shows the core components of a porous electrospray emitter along with three common types of emission.

To increase thrust density, electrospray emitters can be arranged into densely packed arrays. Courtney showed that porous wedge emitters could produce numerous emission sites from a single emitter [9]. Using porous wedge emitters can produce comparable results to conical emitter arrays, while reducing complexity and manufacturing time [10]. The pressure drop between emission site and the porous bulk for a wedge emitter in Eq. 1 [11].

1 $$\begin{aligned} \Delta P_{wedge} = \frac{\mu Q_{site}}{\kappa \theta _{wedge}} \left( \frac{1}{\lambda } ln \left( \frac{R_2}{R_1} \right) - \frac{1}{2 \pi R_2} \frac{K_0 \left(\frac{2 \pi R_2}{\lambda }\right)}{K_1 \left(\frac{2 \pi R_2}{\lambda }\right)} \right) \end{aligned}$$

where, $r_t$ is the radius at which the conical structure becomes spherical, $Q_{site}$ is the flow rate at an emission site, $\theta _{wedge}$ is the specified angle of a porous wedge, based on the upstream radius ($R_1$) and the downstream radius ($R_2$), $\lambda$ is the distance between each emission site along the apex of a porous wedge, and $K_0$ and $K_1$ represent the Bessel function of the second order. The permeability of a porous substrate can be estimated using the Kozeny-Carman equation [12]. The equation considers a material’s effective particle diameter ($D_{eff}$) and porosity ($\phi _p$) as seen in Eq. 2. Porosity is a measure of the void space within a material from a scale of 0-1.

2 $$\begin{aligned} \kappa = \frac{D^2_{eff}}{180} \frac{\phi ^3_p}{(1 - \phi _p)^2} \end{aligned}$$

**Fig. 2:**
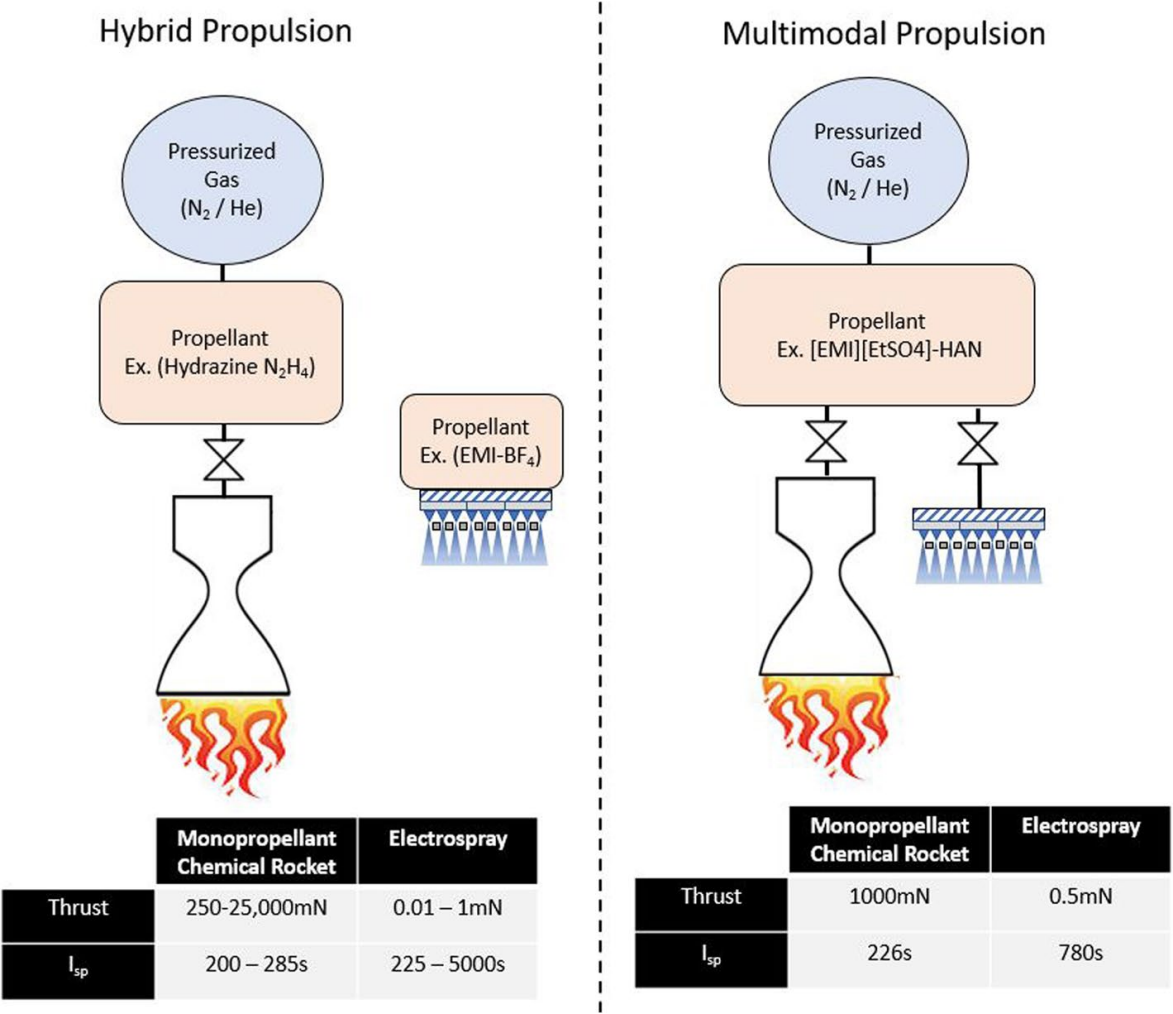
Comparison between a theoretical hybrid, and multimodal propulsion system integrating a monopropellant chemical thruster, with an electrospray [15, 23]

The currently available forms of space spacecraft propulsion (chemical rockets, cold-gas thrusters, EP) each have their strengths and weaknesses. There is a growing interest in developing ways to combine the strengths of high thrust and high $I_{sp}$ modes into a unified propulsion system. Earlier work in the research group had explored both a two-stage ion thruster [13] and a bimodal thruster (combining a cold-gas thruster with an electrospray), the latter demonstrated higher thrust but comes at the cost of a dual propellant system: compressed gas and ionic liquid [14]. Multimodal propulsion is the integration of two or more propulsion modes into a system which utilizes a common propellant source [15]. The concept is shown in Fig. 2. On smaller spacecraft, where physical space comes at a much higher premium, it is ideal to combine many propulsion modes into a single unified system. Having multiple modes of operation can greatly expand the possible missions a satellite can undertake. This means additional adaptability in orbit, and also lowers research and development costs, since a single multimodal system can be used in many mission types. Two identical satellites could be launched into orbit and perform different missions depending on how propellant is used. Several multimodal thrusters have been investigated at various research institutions [16–22].

In this paper we outline a novel concept for a multimodal electrospray thruster incorporating an ion mode electrospray thruster with a droplet mode electrospray thruster using the common IL propellant EMI-BF$_4$. The system allows for a the selection between a high thrust and high efficiency form of propulsion while maintaining the small form factor and good vacuum properties of electropsray thrusters. The concept is aligned with the ongoing trend of satellite miniaturisation. Interestingly, the potential utility of a multimodal electrospray thruster to provide propulsion for small spacecraft should be highlighted. Indeed, spacecraft mission simulations and models have shown that this type of propulsion (multimode) is exciting because of the flexibility and adaptability it provides mission designers and planners. A single spacecraft would have potential to execute drastically different mission profiles, and the exact mission could even be determined post-launch [24].

## Multimodal electrospray thruster design

**Fig. 3:**
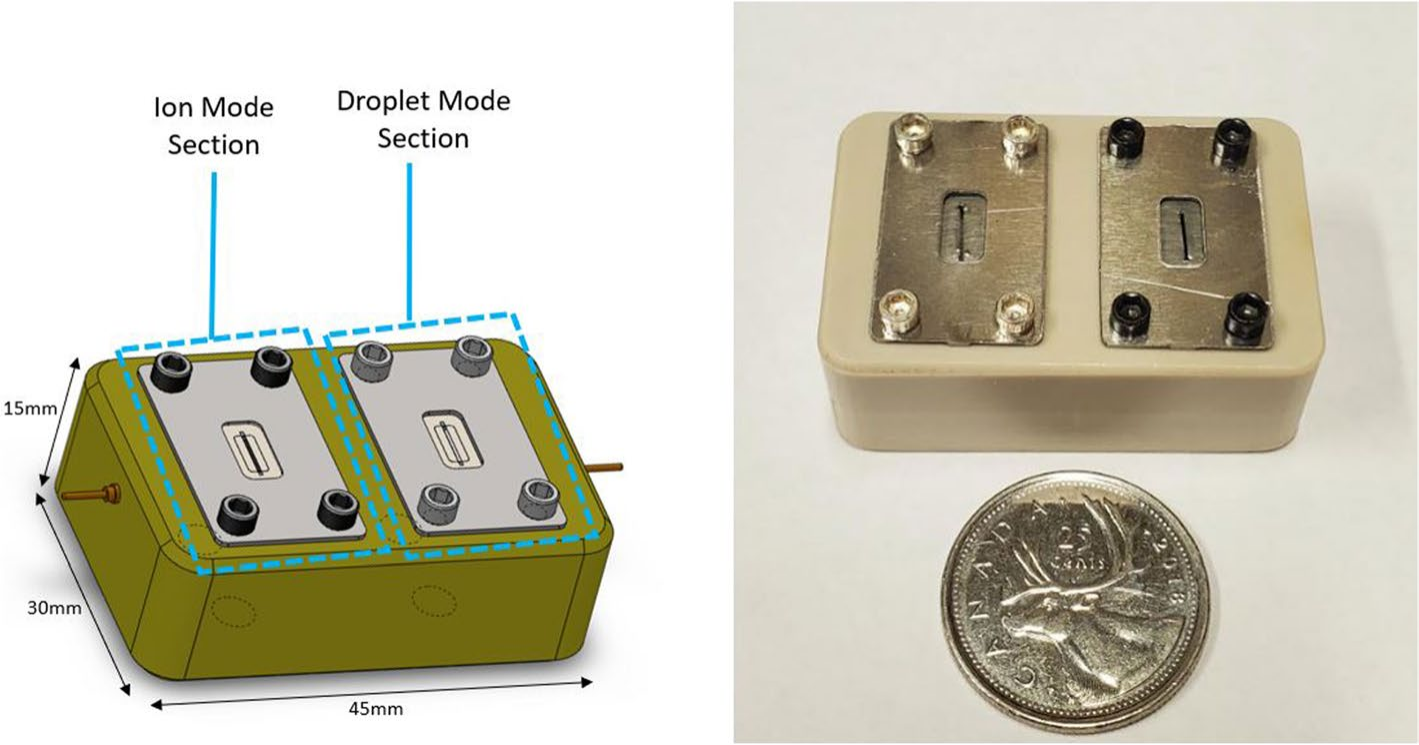
The exterior components of the assembled multimodal electrospray prototype thruster. The LHS shows the CAD model of the thruster and RHS shows the final thruster assembly next to a Canadian quarter (diameter of 23.88mm) for size comparison

The core components of the multimodal thruster are two porous wedge emitters of varying permeability and mean pore size. The variance in pore properties impacts the propellant flow rate and thus the characteristics of electrospray emission. One emitter was desgined to emit purely droplet for high relative thrust, and the other purely ions for high specific impulse. Using only porous electrospray emitters in the design allowed for consistency in both modes of operation. RAPPEL has previously developed several designs of electrospray propulsion systems [14, 25–27]. The presented design was also inspired by several recent electrospray prototypes [9, 10, 28, 29]. The multimodal design is depicted in Fig. 3. As a proof-of-concept, the multimodal thruster is a demonstration of how emission from the two different modes could be integrated into a single system.

The outer dimensions of the thruster are 30 mm x 45 mm x 15 mm. A polyether ether ketone (PEEK) plastic block was used as housing for all thruster components, as it is an insulator with excellent high vacuum properties [30]. The housing was spilt into two sections as seen in Fig. 3, where one section produce an ion mode emission, while the other section a droplet mode emission. The two electrospray emitters have porous wedge geometry. Each emitter had a height of 0.3 mm, a length of 4 mm and were designed to have an apex curvature radius of 10 microns. The emitters were positioned on top of a rectangular alignment platform 0.3mm high milled into the emitter substrates.

The ion mode emitter was fabricated from a porous Carbon Xerogel substrate fabricated in-house, via a slightly adapted process outlined by Arestie [31]. The Carbon Xerogel samples were analyzed in order to determine their mean pore size and porosity. Many produced samples were finely polished and analyzed using scanning electron microscope (SEM) along with the image processing software ImageJ [32]. The Carbon Xerogel samples were determined to have an average pore size of  1.2 microns. The pore sizes did show some minor variation between samples and ImageJ has been known to introduce error into analysis but is able to provide valuable insights. Future work will include more detailed cross-sectional photos for enhanced accuracy and will be very useful in defining wedge emitter strategies. For demonstrating the feasibility of the multimodal thruster the relative pore size determination between the samples was deemed sufficient and the goal of producing a finely porous emitter substrate was achieved for the current objective.

**Fig. 4:**
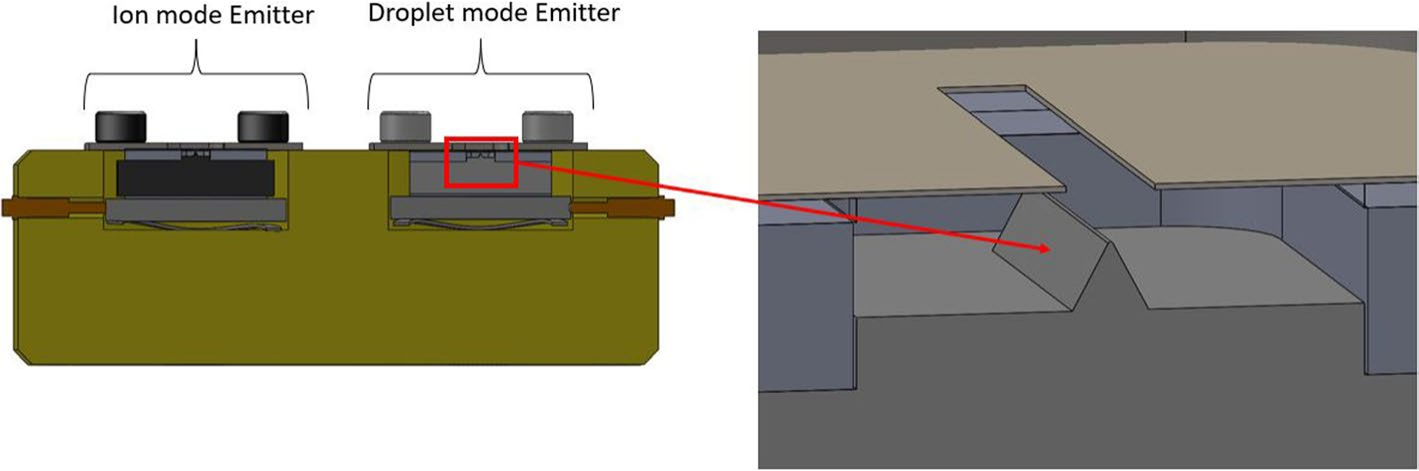
Cross-sectional schematic of the designed multimodal thruster

The droplet mode emitter substrate was a commercially purchased P3 porous borosilicate glass filter with a mean pore size of  40 microns. The larger pore sizes of the borosilicate glass emitter created an increase to the propellant flow rate allowing for droplet emission. The small pores of the Carbon Xerogel substrate inhibited propellant flow allowing for ion emission. The extractor electrodes made from 0.05mm thick molybdenum foil were separated from the electrospray emitter by a PEEK spacer. The extractor and PEEK spacers were attached using double sided kapton tape. By pressure fitting the PEEK spacer to the emitter alignment platform it allowed for automatic alignment of the emitter apex and the extractor electrode during assembly. The distance from the apex of the electrospray emitters to the extractor electrodes was designed to be 0.3 mm. A cross sectional view of the emitter, extractor, and alignment platform can be seen in Fig. 4.

Stainless steel porous discs were used as both distal electrodes and propellant reservoirs for the electrospray emitters. The ion mode emitter was coupled with a stainless steel disc that had a porosity of 28 - 32% and pore rating of   5 microns. A pore rating measures the diameter of particles which the porous material would not allow to pass through. The droplet mode distal electrode was a disc with a porosity of 55 - 65% and pore rating of  40 microns. The porous properties of the distal electrode have an impact on electrospray emission. Each distal electrode was selected so that there would be minimal impact to propellant flow rates through the emitter substrate [9]. A single piece of filter paper was placed between the distal electrodes and the electrospray emitters to ensure that the components were hydraulically coupled during operation. Two small holes were drilled into the sides of the PEEK housing and were aligned to the edge of the distal electrodes. After the internal components were assembled, a pogo pin electrical connection was pressure fitted into the holes, permitting for the high voltage power supplies to be in contact with the distal electrodes.

### Predicted results

The electrospray propulsion engineering toolkit (ESPET) [33] was used to estimate the predicted performance of electrospray emitters. The software considers key physical factors such as emitter and reservoir material, emitter geometry, emitter properties, as well as the propellant used. Known parameters of the multimodal thruster were input to the ESPET quick solver tool. Electrospray thrusters, like many forms of EP have the potential to throttle their output - and throttling the applied voltage can have a large impact on the performance parameters such as thrust, specific impulse, and current. ESPET provided the estimated outputs over a range of applied voltages. To maintain consistency for estimates, all output parameters were calculated at a common applied voltage of 1,750 V. The expected results for the multimodal thruster are shown in Table 1. The predicted performance estimates of the designed multimodal thruster from ESPET were used as a baseline for the experimental results. It should be highlighted that in recent work [27] discussed that ESPET [33] and wedge modeling [34] can display some disparity in important parameters such as starting voltage, specific impulse and thrust. This in turn complicates direct comparisons of theoretical to experimental values as they are dependant upon physical parameters such as number of emission sites on the wedge or beam composition of ions to droplet ratio, but is helpful as it does provide an order of magnitude for expected results.

**Table 1:** Predicted multimodal performance using ESPET at an applied voltage of 1750V

	Thrust ($\mu N$)	Specific Impulse (*s*)	Current ($\mu A$)	Mass Flow Rate ($^{\mu {g}/}\!\!/_{s}$)
*Ion Mode*	0.33	1865	3.68	0.0178
*Droplet Mode*	543	123	275	448

## Fabrication and assembly of prototype

**Fig. 5:**
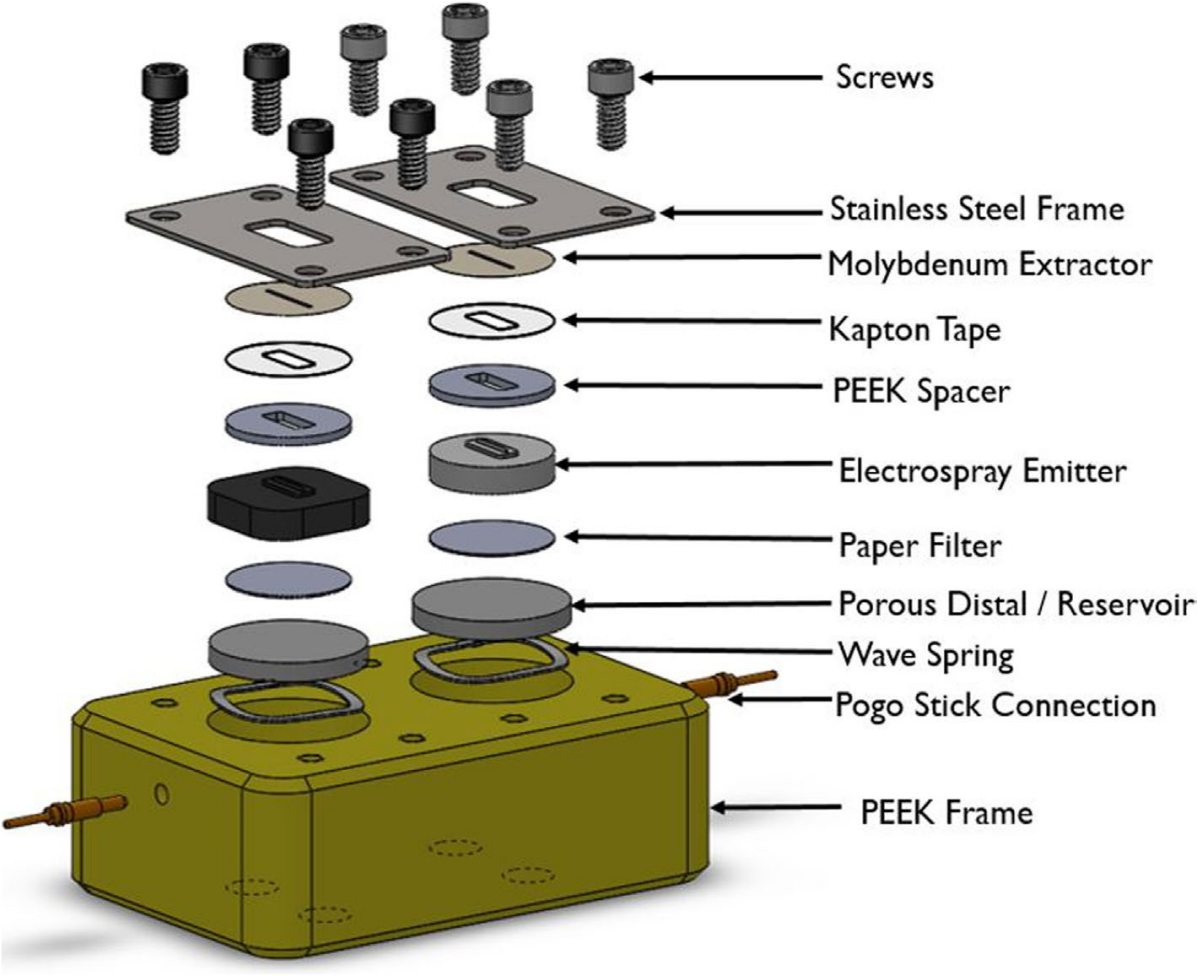
Exploded view of internal multimodal thruster components

The various internal components which constitute the thruster is shown in Fig. 5. Several of the components were COTS but the PEEK frame, stainless steel frame, and PEEK spacer were machined in-house. Laser milling was used to etch wedge emitters and alignment platforms out of the Carbon Xerogel and borosilicate glass materials. Laser milling was also used to cut the extractor electrodes and was performed at Nanofabrication Kingston (NFK) by an Oxford Picosecond Laser Series A.

**Fig. 6:**
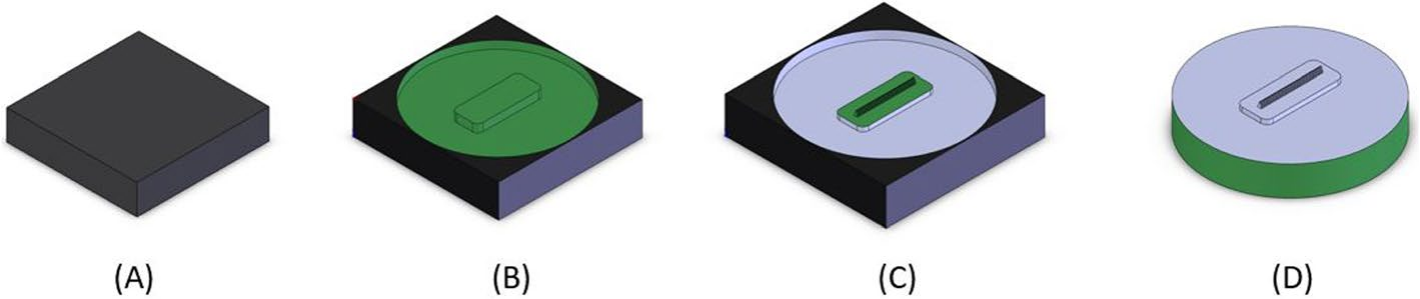
The steps taken to fabricate the Carbon Xerogel electrospray emitter. **A** Carbon Xerogel sample, **B** Alignment platform laser milled, **C** Emitter laser milled through a series of 25 laser passes, **D** Excess material is sanded away to circular shape

Laser ablation is a process by which a high-power laser is used to essentially melt and evaporate solid material [35]. Since the energy of the laser is concentrated to a small area, usually microns in size, it can remove layers of material without impacting the porous structure below. Laser ablation has been used in several past designs for emitter fabrication in the literature [14, 36, 37] The steps taken during Laser milling of the emitters is shown in Fig. 6. Each emitter substrate had the alignment platform a depth of 600 microns. Next the wedge emitters were milled by a series of 25 Laser passes at low power. Using lower Laser power was less efficient, but decreased the potential for a samples’ pores being fused together [38]. Each of the 25 laser passes were rectangular with a length of 400 microns, and width varying from 10 to 250 microns. The 25 passes created a series of steps which closely resembled a 3D wedge emitter shape.

**Fig. 7:**
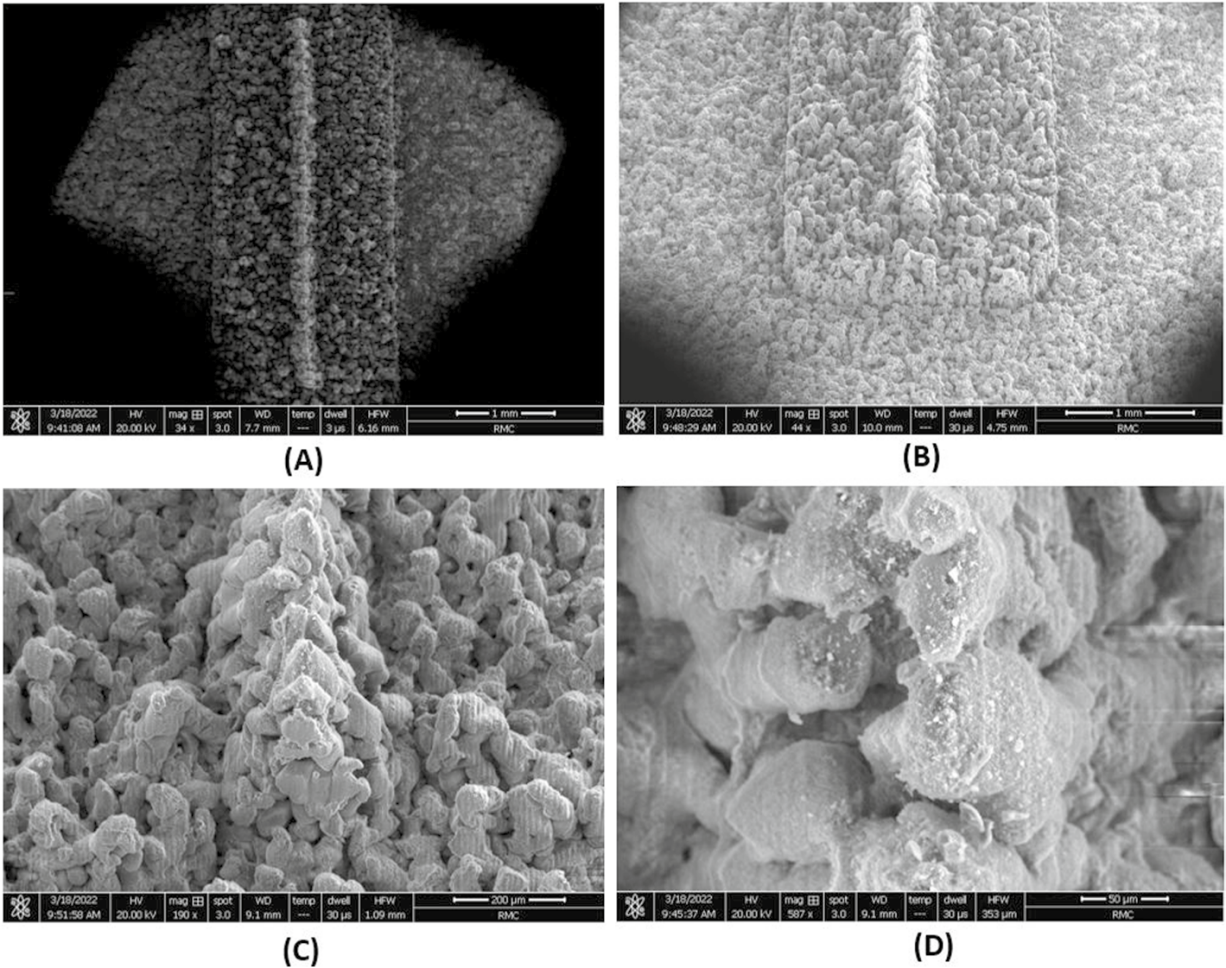
SEM imagery of fabricated P3 borosilicate glass wedge emitter. **A** Top view of emitter and platform (magnification 34x), **B** Enhanced view of emitter and platform (magnification 44x), **C** One side edge view of the wedge emitter (magnification 190x), **D** View of wedge emitter apex (magnification 587x)

**Fig. 8:**
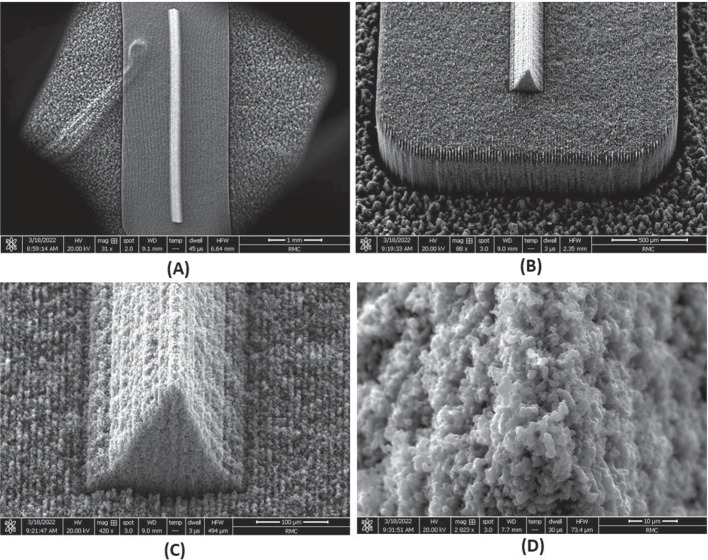
SEM imagery of fabricated Carbon Xerogel wedge emitter. **A** Top view of emitter and platform (magnification 31x), **B** Enhanced view of emitter and platform (magnification 88x), **C** One side’s edge view of the wedge emitter (magnification 420x), **D** Emitter apex view (magnification 2823x)

Inital test samples with the Laser were analyzed using a Bruker DektakXT Stylus Profilometer to determine the ablation rate of the emitter substrates. For glass droplet mode emitters the alignment platform was milled using a series of 4 passes at a 100% power setting and mill rate of 0.5 mm/s. The laser beam width at this power setting was 65 microns. Before each laser pass, the sample was auto-focused so that the Laser was focused to the correct depth. Next, the emitter was milled in a series of 25 laser passes at a power setting of 13% and mill speed of 1 mm/s. Images of an example emitter using an SEM are shown in Fig. 7. The Carbon Xerogel ion mode electrospray had their alignment platforms milled in a series of 2 laser passes at a power setting of 100% and mill rate of 0.65 mm/s. Next, the emitter was milled in a series of 25 laser passes at a power setting of 3% and mill speed of 1 mm/s. After the emitter was formed, the outside edges were sanded by hand, to give the sample the same circular shape as the borosilicate glass. Magnified views of a sample Carbon Xerogel emitter can be seen in Fig. 8.

To fabricate the extractor electrodes, 10 mm circles were Laser cut from a sheet of molybdenum foil using 10 passes of the laser at a power setting of 100% and milling speed of 0.5 mm/s. Each of the circles were then adhered to a PEEK spacer using double-sided Kapton tape. The extractor assembly was then put back into the Laser machine to have the extractor slit cut out. The slit was a rectangular hole 5 mm in length and 0.35 mm in width. The Laser was aligned to the center of the molybdenum foil, and the slit was cut using the same laser parameters as the molybdenum circle cuts.

## Experimental testing setup

All experimental tests performed for this research were conducted at the Royal Military College Advanced Propulsion and Plasma Exploration Laboratory (RAPPEL). A bimodal thrust stand was previously developed at RAPPEL [38] which used two different propellants. The stand is capable of direct thrust measurement (DTM) in the millinewton (mN) range, using torsional balance, and indirect thrust measurement (ITM) to collect current from charged particle beams using a metallic collector plate. This work builds upon the ITM system to allow for ToF measurements of electrospray thrusters. The pre-existing thrust stand was used as a base on to which components were added to create the diagnostic ToF system.

### Vacuum chamber

**Fig. 9:**
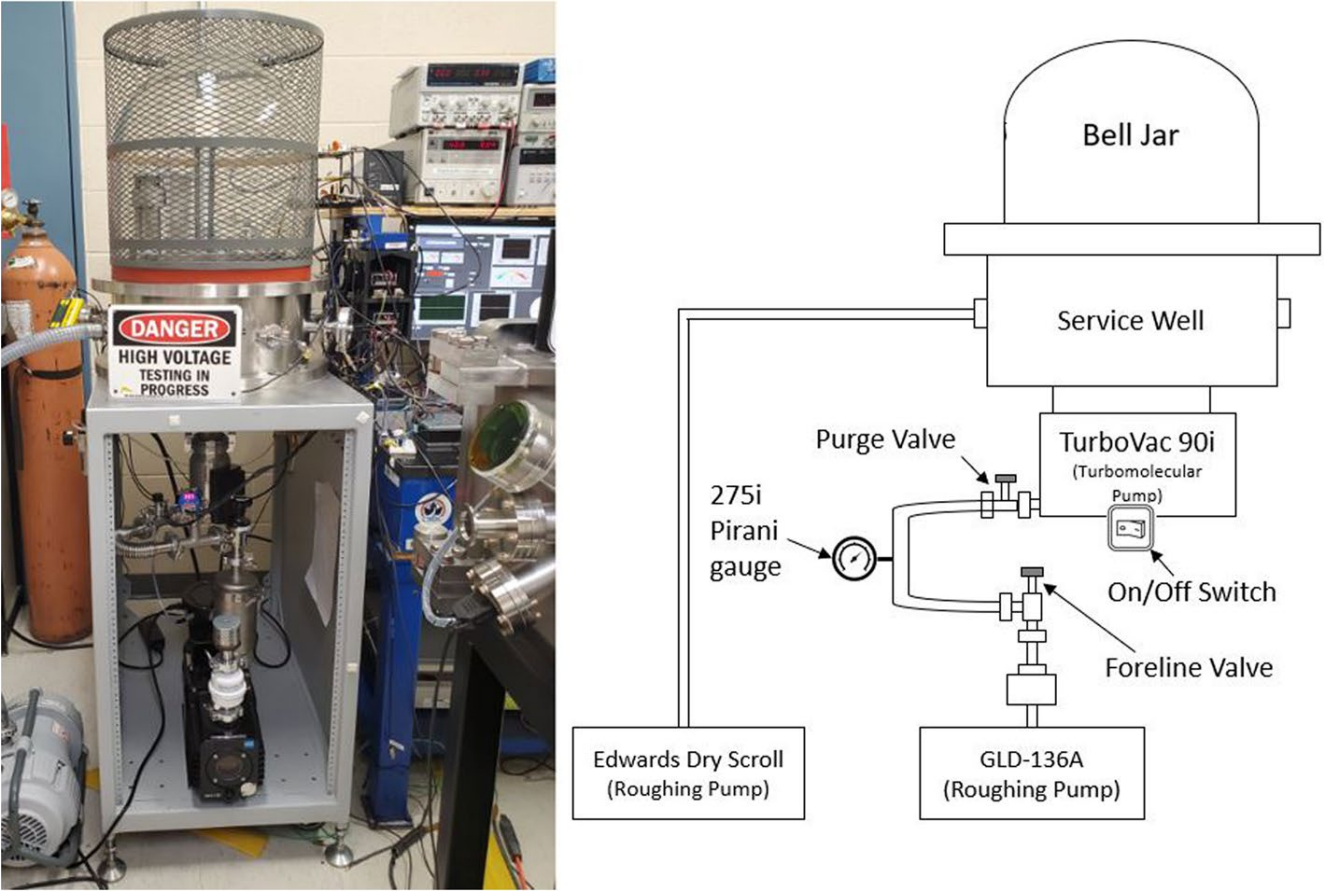
Bell Jar Vacuum Chamber used for experimental testing: two roughing and one turbo-molecular pump used to bring the chamber to a minimum measured pressure of 5x10$^{-6}$ Torr

To replicate the conditions of space as best possible, high vacuum pressure chambers are used at RAPPEL: a custom bell jar chamber built by the Kurt J. Lesker, as shown in Fig. 9, was used. The chamber was brought to high vacuum pressure using a two-stage vacuum pump system, which included a ULVAC Technologies Inc. GLD-136A and Edwards Vacuum XDS10 dry scroll roughing pumps connected in series with a Leybold TURBOVAC 90i turbo-molecular pump. The combination of pumps allowed the chamber to reach a vacuum state of between 8x10$^{-6}$ to 1.5x10$^{-5}$ Torr achieved after on average 2.5 hours of vacuum operation.

Pressure within the vacuum chamber was measured using a Superbee CVM201 pressure gauge and a Kurt Lesker company 354 Series hot cathode ionization gauge. The 90i turbopump and the GLD-136A roughing pumps were connected to the primary vacuum port at the base of the bell-jar chamber. The chamber also had eight input/output connection ports around its outside surface. These ports were used to connect pressure gauges, the Edwards dry scroll roughing pump, and various voltage/current input and output signals.

Even at these low pressures the charged particle beam produced during experimentation interacted with the remaining residual gases inside the vacuum chamber. These interactions have been shown to increase beam emittance and reduce its overall strength [39]. To allow for consistency testing was conducted in the same pressure range for all the setups.

### Prototypes testing setups

**Fig. 10:**
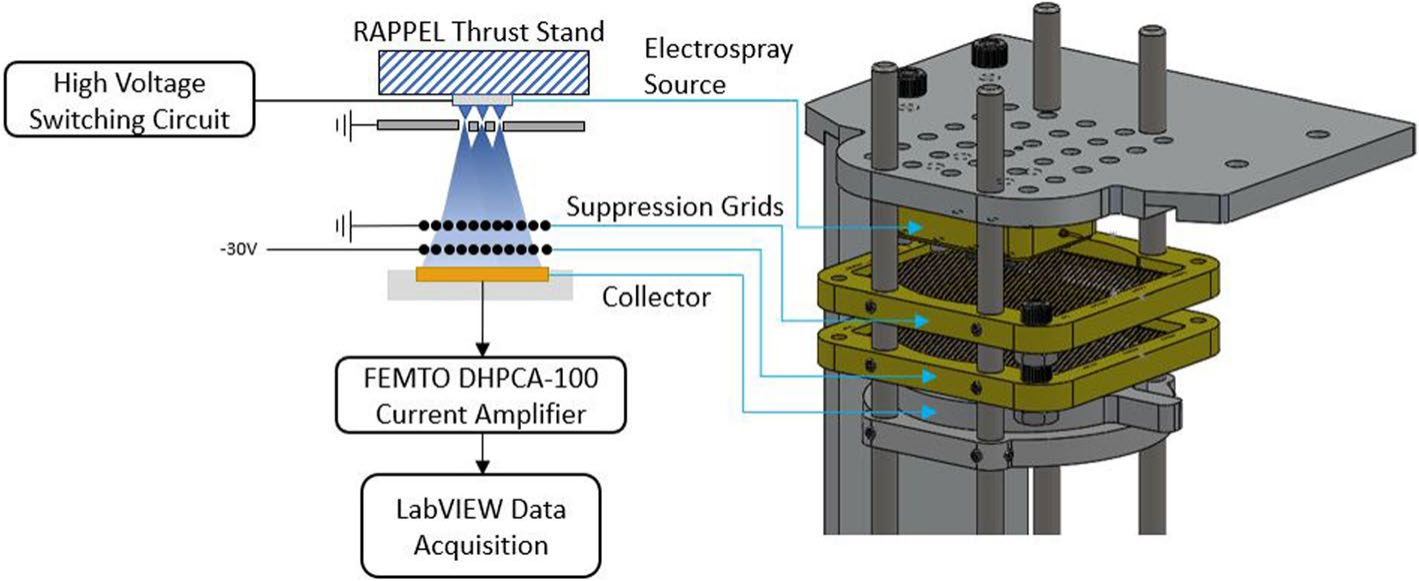
Experimental setup used to collect full beam current measurements from electrospray source

Thrusters producing particle beams are commonly tested using both direction and indirect methods [40, 41]. Here the tested electrospray propulsion prototypes were characterized indirectly using two experimental configurations: a full beam setup, and a time-of-flight (ToF) setup. The purpose of full beam measurements was to collect the entire emitted charged particle beam of the selected electrospray source. The charged particles produced by an electrospray carry a current characterizing a thruster’s output. In this configuration, an electrospray source was placed near a metallic collector plate with two suppression grids. As charged particles collided with the collector, the strength of the emitted current was measured. A schematic giving an overview of the full beam measurement setup can be seen in Fig. 10.

The collector used in this work was a circular flat stainless steel plate with a diameter of 29 mm. The collector assembly was manufactured in-house during previous work at RAPPEL [38]. In order to compensate for the secondary electrons released at the collector during operation, two suppression grids were placed directly upstream of the collector. The grids were comprised of a PEEK frame with forty-nine 0.025mm diameter fine tungsten wires strung across an open area. The wires were weaved through the PEEK frame by hand using a microscope. Only 2.45% of the frontal area was blocked by the wires, allowing the majority of electrospray emission to pass through uninhibited. The first grid was biased to ground while the grid closest to the collector was biased to -30 V.

**Fig. 11:**
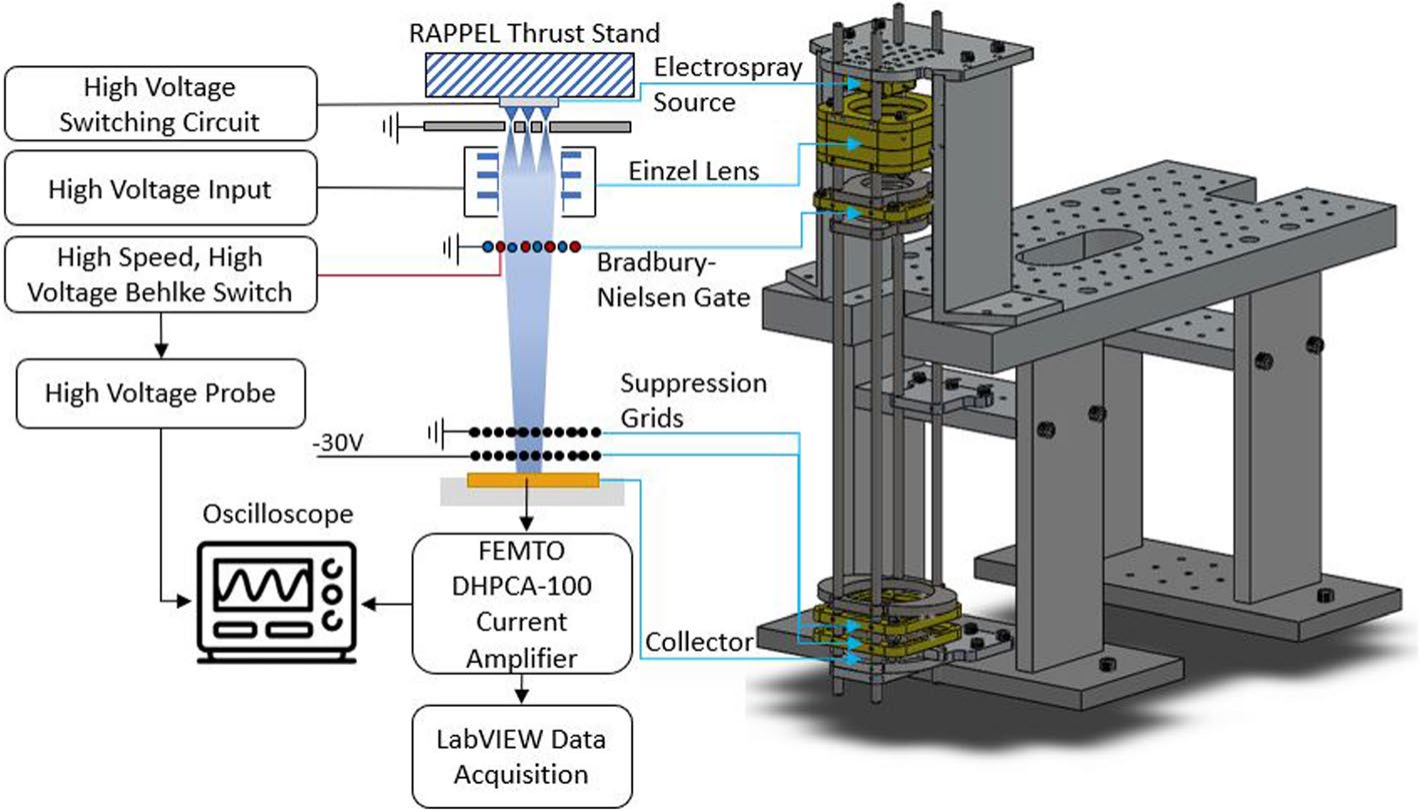
Experimental setup used to collect time-of-flight data from electrospray source

The low absolute thrust produced by electrospray thrusters has led to challenges using direct thrust measurement (DTM) testing methods. Indirect thrust measurement (ITM), using the time-of-flight (ToF) method, is commonly used as an alternative to DTM of electrospray thrusters [10, 42, 43]. The ToF setup shown in Fig. 11 contains a longer flight path, a Bradbury-Nielsen gate (BNG) and Einzel lens, in addition to the components from the full beam setup.

The ToF method measures the exit velocity ($v_e$) of charged particles by measuring the time (*t*) taken to traverse and known flight length distance (*L*) seen in Eq. 3. Once the velocity of the beam has been determined, the $\frac{q}{m}$ of the charge per unit mass can be found using Eq. 4.

3 $$\begin{aligned} v_e = \frac{L}{t} \end{aligned}$$

4 $$\begin{aligned} \frac{q}{m} = \frac{v_e^2}{2 \phi _B} \end{aligned}$$

In order to complete this calculation, the beam potential ($\phi _B$) must be known. In most applications, it is likely that only the voltage applied to the thruster emitters ($\phi _A$) will be known. The $\phi _B$ and the $\phi _A$ are closely related, but not identical. This is due to electrospray loss mechanisms which reduce the overall energy of the particle beam.

Equations 3 and 4 can be rearranged to determine the theoretical flight time of a known ion or droplet with a known mass / charge as shown in Eq. 5. If we assume the beam potential is known, then the mass flow rate, thrust and specific impulse can be indirectly calculated using Eqs. 6, 7, 8.

5 $$\begin{aligned} t_{theo} = \sqrt{\frac{L^2}{2\phi _B}\frac{m}{q}} \end{aligned}$$

6 $$\begin{aligned} \dot{m}_i = I_B f_i \frac{m_i}{q_i} \end{aligned}$$

where $I_B$ is the beam current measured at the collector, $f_i$ is the current fraction produced by an individual species of ions or droplets. The total mass flow rate is the sum of the flow rates of each particle species emitted in the beam.

7 $$\begin{aligned} T = \Sigma (\dot{m_0} v_{e0} +\dot{m_1} v_{e1} + ...) \end{aligned}$$

8 $$\begin{aligned} I_{sp} = \frac{T}{\dot{m}_T g_0} \end{aligned}$$

where $I_B$ is the beam current measured at the collector, $f_i$ is the current fraction produced by an individual species of ions or droplets. The total mass flow rate is the sum of the flow rates of each particle species emitted in the beam. $\dot{m}_T$ is the total mass flow rate. The velocity of a particular charged particle is based on its mass-to-charge ratio. If charge remains constant, heavier particles will take longer to travel a set distance. The use of a ToF system allows for the accurate measurement of the thrust and specific impulse of electrospray emitter.

A stainless steel stand was added to the pre-existing RAPPEL thrust stand [38] in order to increase the flight distance between the electrospray source and the collector. A longer flight distance leads to increased flight times, allowing for increased resolution for the current readings at the collector. During ToF testing the measured flight distance was 33 cm.

**Fig. 12:**
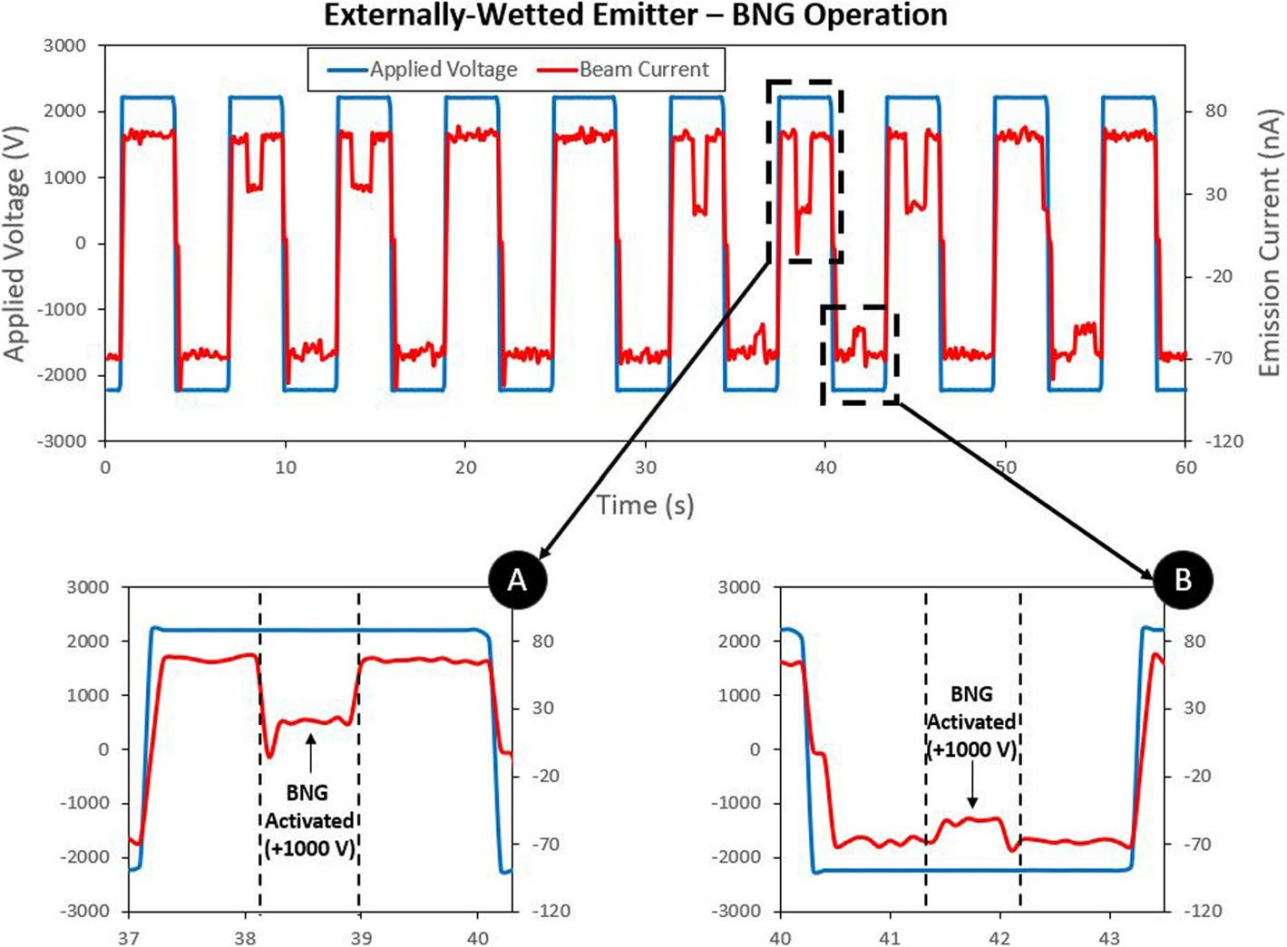
BNG testing results using a single externally-wetted emitter. Device consistently deflected the majority of positive ions

A Bradbury Nielsen gate (BNG) was selected to deflect the particle beam in the ToF setup. The BNG was converted from a pre-existing suppression grid used in previous work at RAPPEL [38]. Ninety-eight (98) strands of 0.025 mm diameter tungsten wire were strung through a PEEK frame. Small holes drilled into the PEEK frame were spaced by 0.4 mm to create wire separation. Adjacent wires were isolated so that alternating wires could be biased to different voltages. The tungsten wire blocked 5% of the frontal area, allowing the majority of the particle beam to pass through undisturbed. A sample preliminary test of the BNG shown in Fig. 12. The BNG was consistently successful in deflecting the majority of the positive ion beam emission, but was less effective with negative ions. The presented time-of-flight results of the multimodal thruster focus on positive ion emission.

**Fig. 13:**
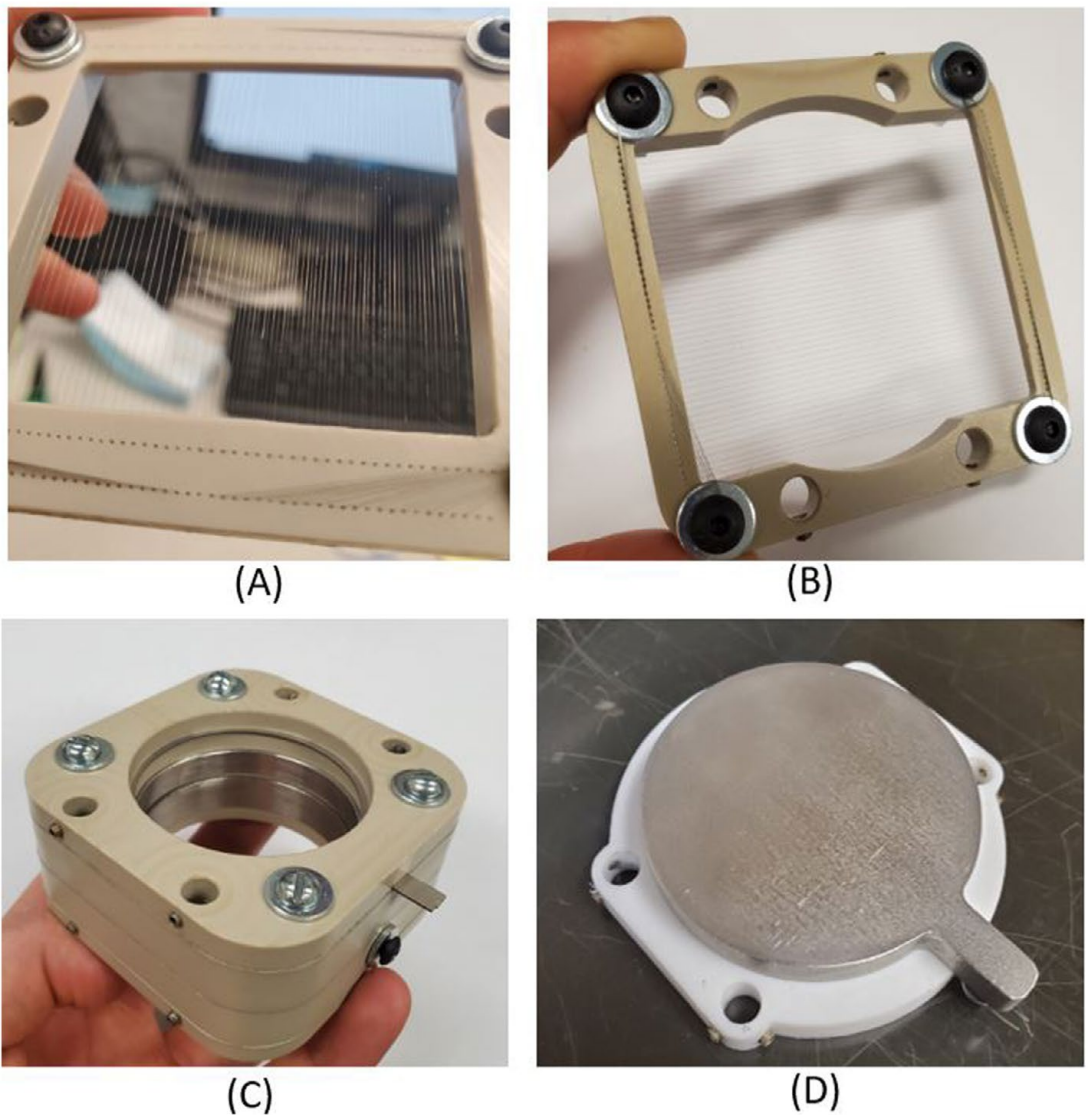
The various components which comprise the ToF system. **A** Bradbury-Nielsen Gate, **B** Suppression grid, **C** Einzel Lens, **D** Beam Collector

An Einzel lens was designed to be easily integrated into the RAPPEL thrust stand. While not critical to operate a ToF system, the lens was a useful supplementary tool for focusing electrospray emission over a large distance as described in [44]. The asymmetric lens was comprised of three cylindrical stainless steel electrodes spaced apart using a PEEK frame. It is interesting to note that while the Einzel Lens was successfully tested, the presented results below did not use the device since only certain charge-to-mass ratio ions would be focused. The BNG, Einzel lens, suppression grid a collector are depicted in Fig. 13.

### Electrical connections

**Fig. 14:**
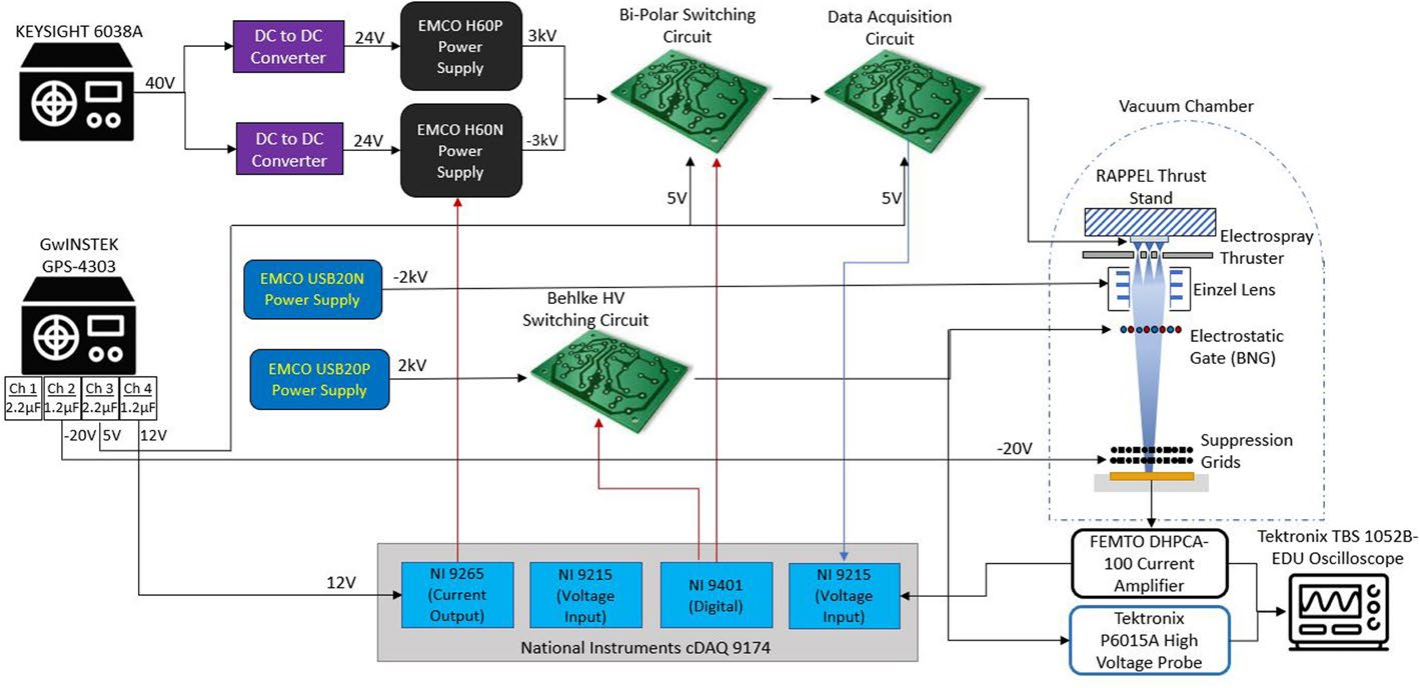
Overview of the power system and circuitry used to test electrospray thrusters

The circuitry, power supply, and data acquisition components used to operate the electrospray thrusters and the associated testing apparatus are summarized in Fig. 14. The tested electrospray sources were supplied high voltage by two EMCO H60 (0-6kV) power sources. The H60 units supplied both positive and negative potentials to the electrospray. Bipolar voltage operation was achieved by using a square wave signal from a LabVIEW control program. A switching frequency of 0.333 Hz was used to ensure there was enough time to make ToF readings, while negating electrochemical effects of single polarity operation. The output of the bipolar switching circuit was connected to a data acquisition circuit board, which measured the voltage and current applied to the electrospray source being tested.

The collector plate was connected to a FEMTO DHPCA-100 series variable-gain high-speed current amplifier. The selected amplifier gain setting varied from 10$^4$ to 10$^8$ volts per ampere of current depending on the mode of operation being tested. The amplifier is also capable of operating at a collection frequency of 10 MHz, allowing for measurements on the scale of micro-seconds. A National Instruments data acquisition system was used to output control signals and measure input signals from the electrospray system. The testing apparatus and electrical connections is also discussed in [45].

### Testing procedure

The assembled multimodal thruster was fastened to the ToF stand and the vacuum chamber was brought down to pressure. The voltage applied to the thruster was increased from 0 V to a maximum of 3,000 V in 25 V increments a few seconds at a time. The voltage was raised until the first sign of emission. Data was then collected, based on the particular test which was being performed. The multimodal thruster was tested first in the full beam configuration, followed by a ToF configuration for both droplet and ion mode.

## Results and discussion

The ion and droplet emitters which comprise the multimodal thruster described in this paper were each tested independently. Initial testing led to changes in the thruster’s design which will be elaborated in the discussion section. Results using this final configuration are presented in this section. Full beam testing of both emitters demonstrated a measured current range in line with expected results. ToF measurements allowed for the distinction of various ion and droplet species. Using data collected from ToF testing, indirect values for the mass flow rate, thrust, and specific impulse were calculated for each of the modes as discussed in the next section. The relative limitations which can affect the results will be highlighted and is accounted for while analyzing the results, for instance in cased where unstable beam emission were observed at higher applied voltages. The high signal-to-noise ratio due to limitations of measurement requirements made it difficult to identify droplet species. Hence beyond the ion spectrum the measurement quality has to be kept in mind as droplets and higher species could still be present but not be detected by the current setup. The calculated performance parameters used full beam and ToF data from separate experiments of the same emitter. The performance parameters which were derived from the data should therefore be viewed in the context of the experiments limitations.

### Ion mode emitter results

#### Full beam

**Fig. 15:**
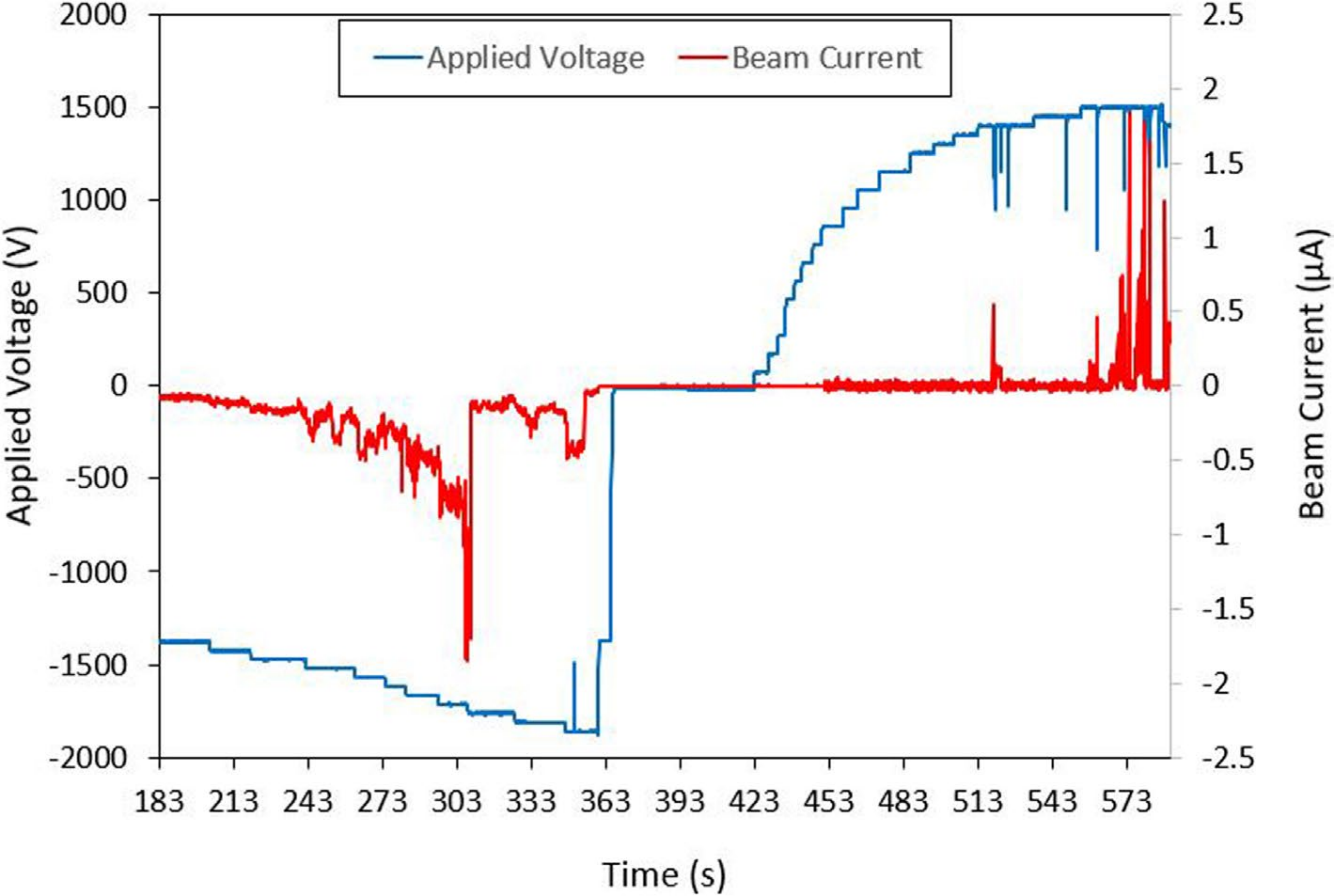
Full beam measurement test results for the Ion mode emitter. Presented test results show unipolar application of voltage where positive voltage is first applied then the negative voltage

**Fig. 16:**
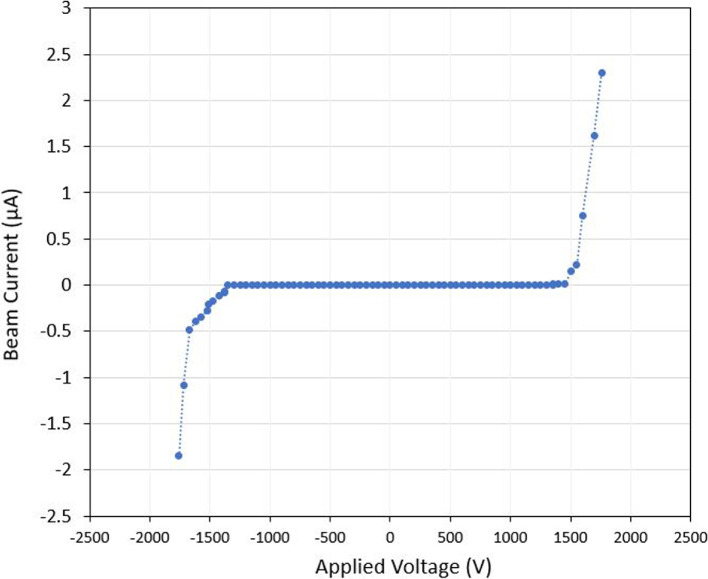
Ion mode emitters current to voltage relationship (I-V Curve)

The ion mode emitter had an average emission onset voltage around 1400 V. A sample unipolar test shown in Fig. 15 had an onset voltage around 1375 V. In this test, the measured current was around +2.3 $\mu$A and -1.85 $\mu$A at an applied voltage of ±1750 V. As the emitter voltage was increased in increments of 25 V, there was a clear correlation in the measured beam current. This relationship between the current and voltage can be seen in I-V curve from Fig. 16. The I-V curve for both the ion and droplet emitter were produced using a set of data points collected when emission was consistent. It should be noted that beam emission at higher voltages was rather unstable leading to some uncertainty in the presented I-V results. Breaks in emission after extended periods due to a build up of charge from single polarity operation were also observed.

#### Time-of-flight measurements

ToF measurements for the positive emission polarity were collected using an oscilloscope and filtered with GNU radio. The ToF data revealed that the ion mode emitter operated in either the purely-ionic-regime (PIR) or near-PIR. During testing a negative spike in current immediately after the BNG was deactivated was followed by the expected step increases. Two current step increases within the first 10 $\mu$s indicate the presence of multiple species of ions. The lack of any noticeable current increases beyond the first 10 $\mu$s indicates that there were no heavier charged particles, such as droplets, within the emitted beam. Based on the flight times of the two current spikes, they were identified as the monomer ion (EMI$^+$), and the dimer ion ((EMI-BF$_4$)EMI$^+$). Testing results of negative ion emission has not been reported on, since the BNG used in the testing set up was ineffective during negative emission ToF testing.

**Fig. 17:**
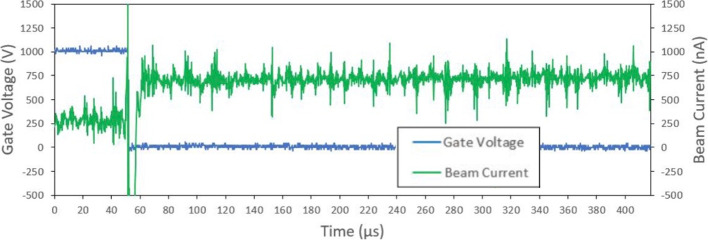
Sample ToF test data for Ion Mode emitter in positive polarity

**Fig. 18:**
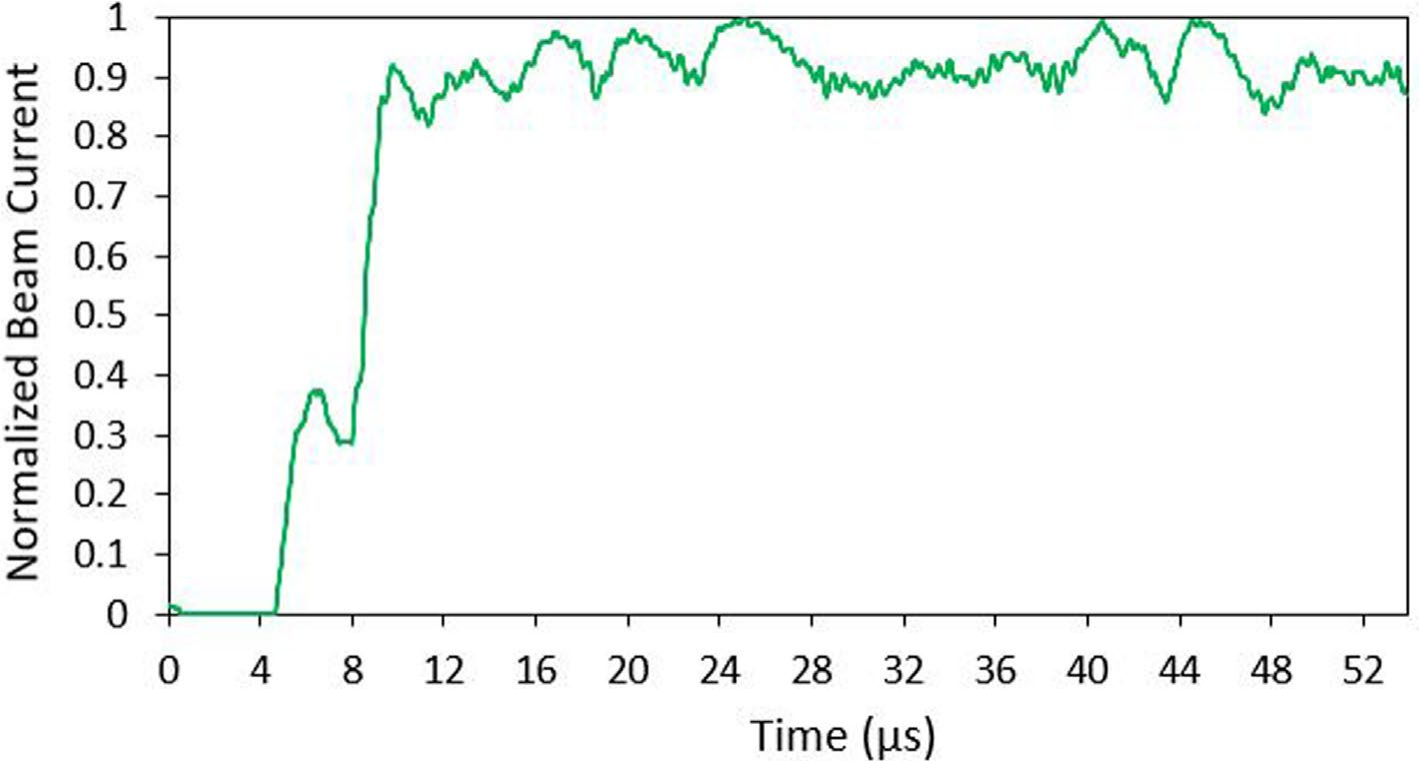
Normalized ToF data. The start of the plot (time = 0) is when the BNG is deactivated. Two distinct currents steps indicates the presence of both monomer and dimer ion species

The total current measured at the collector during ToF testing was on the order of 750 nA as seen in Fig. 17. The current data from the oscilloscope was filtered and normalized in order to determine the percentage of each ion species present in the beam. The current fluctuations considered to be outliers were removed from the data set. This allowed for the actual step changes in current to be easily viewed. A 20 point running average of data points was calculated to smooth the results and the current data was normalized from the point when the BNG was deactivated (t=0). This normalized data is shown in Fig. 18. The normalized data determined that around 42$\%$ of the beam consisted of monomer ions and 58$\%$ were dimers. No large ions such as trimers or liquid droplets were identified in the data (as discussed previously, it could be due to instrumentation limitations).

The flight time and beam composition were used to indirectly determine the mass flow rate, thrust and specific impulse. In order to complete these calculations, several other parameters need to be known. The flight distance was known from the experimental setup. The applied beam voltage and collector current were taken from LabView. The beam voltage was assumed to be equivalent to the applied thruster voltage. With the known values and assumptions, the mass flow rate, thrust, and specific impulse were indirectly calculated using Eqs. 6, 7 and 8. For comparison, the theoretical flight times of the ion species were also calculated using Eq. 5. The thrust of ion mode was determined to be approximately 0.14 $\mu$N, and the specific impulse 4040 s. A summary of the ion mode emitters parameters are shown in Table 2.

**Table 2:** Ion mode emitter performance results summary. Uncertainty based on data variance not including systematic error. Upper limit of Thrust and specific impulse are presented

	Atomic Mass (*amu*)	Expected Flight Time ($\mu s$)	Measured Flight Time ($\mu s$)	Beam Composition ($\%$)	Mass Flow Rate ($^{\mu {g}/}\!\!/_{s}$)	Upper limit of Thrust ($\mu N$)	Upper limit of Specific Impulse (s)
EMI$^+$	111	5.22	6.1 ±0.1	42 ±10	7.62 $\cdot$ 10$^{-4}$		
(EMI-BF$_4$)EMI$^+$	309	8.71	9.2 ±0.5	58 ± 10	2.79 $\cdot$ 10$^{-3}$	0.14	4040
(EMI-BF$_4$)$_2$EMI$^+$	507	11.16	N/A	0	N/A		
Droplets	N/A	N/A	N/A	0	N/A		

### Droplet mode emitter results

#### Full beam

**Fig. 19:**
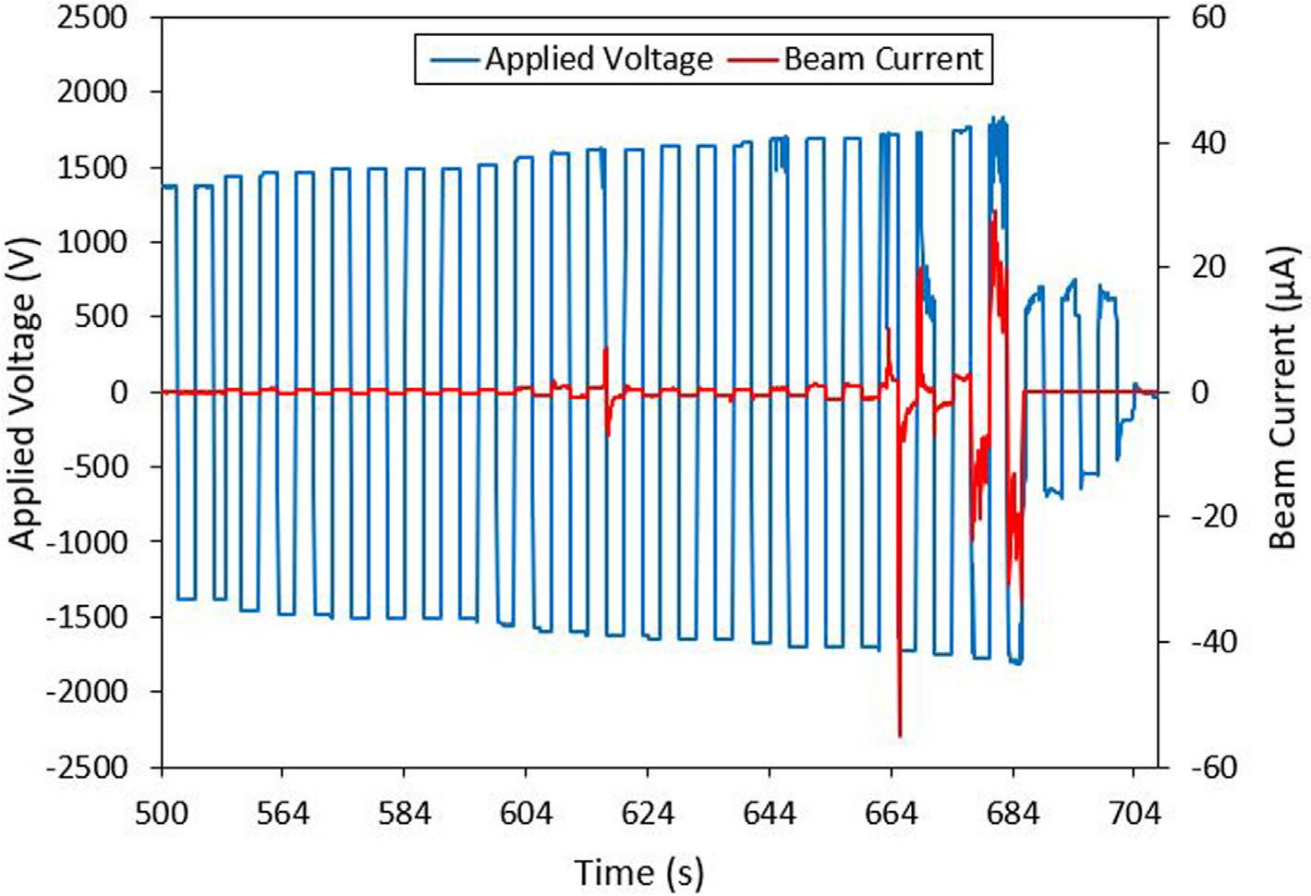
Full beam measurement test results for the Droplet mode emitter. Test used bi-polar operation with voltage polarity alternating every 3 seconds

**Fig. 20:**
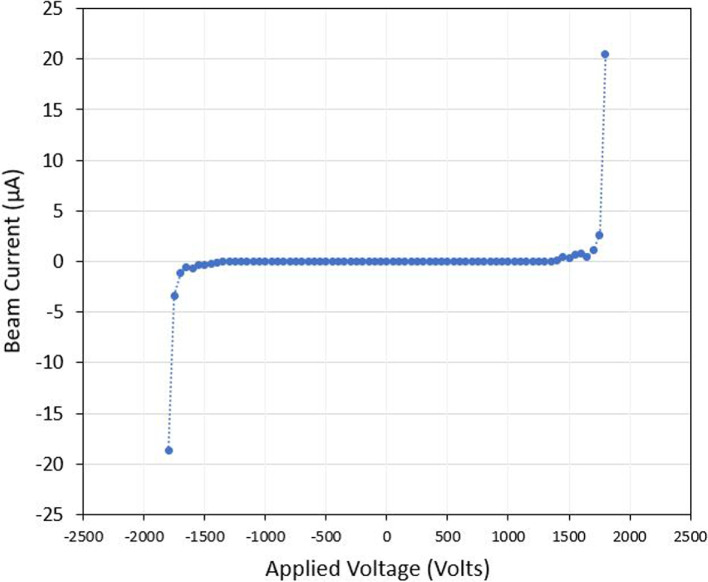
Droplet mode emitter’s current to voltage relationship (I-V curve)

Droplet mode had an onset voltage around +/- 1375 V. Initially, a beam current of around 50 nA was observed. The emitter fired consistently and gradually increased in current output from onset up to  1650 V. After 1650 V, the current output spiked to as high as 80 $\mu$A. In the sample droplet mode test seen in Fig. 19 the emitter had a consistent output of around 0.5 - 1 $\mu$A and then jumped to between +20.5 $\mu$A and -18.7 $\mu$A with current spikes up to -55 $\mu$A. Following the spike in current output, short circuits during emission were more common in the droplet mode emitter as consistent with observations in literature. The trend of applied emitter voltage to output current is shown in Fig. 20. This is a well known phenomenon which is inherent to physical phenomena such as propellant chemical decomposition or flooding [10, 46] and can be attenuated as shown in unipolar tests [47]. The I-V curve was produced with beam emission data which contains unstable sections as discussed previously. The results presented are meant to give a general trend rather than being an absolute representation but do aim to contribute to an insightful description of the global behaviour of the multimodal thruster.

#### Time-of-flight measurements

**Fig. 21:**
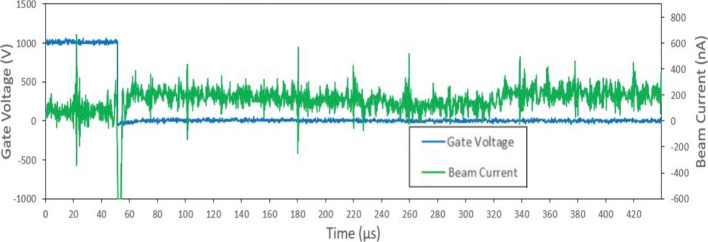
Sample ToF test data for Droplet Mode emitter in positive polarity

**Fig. 22:**
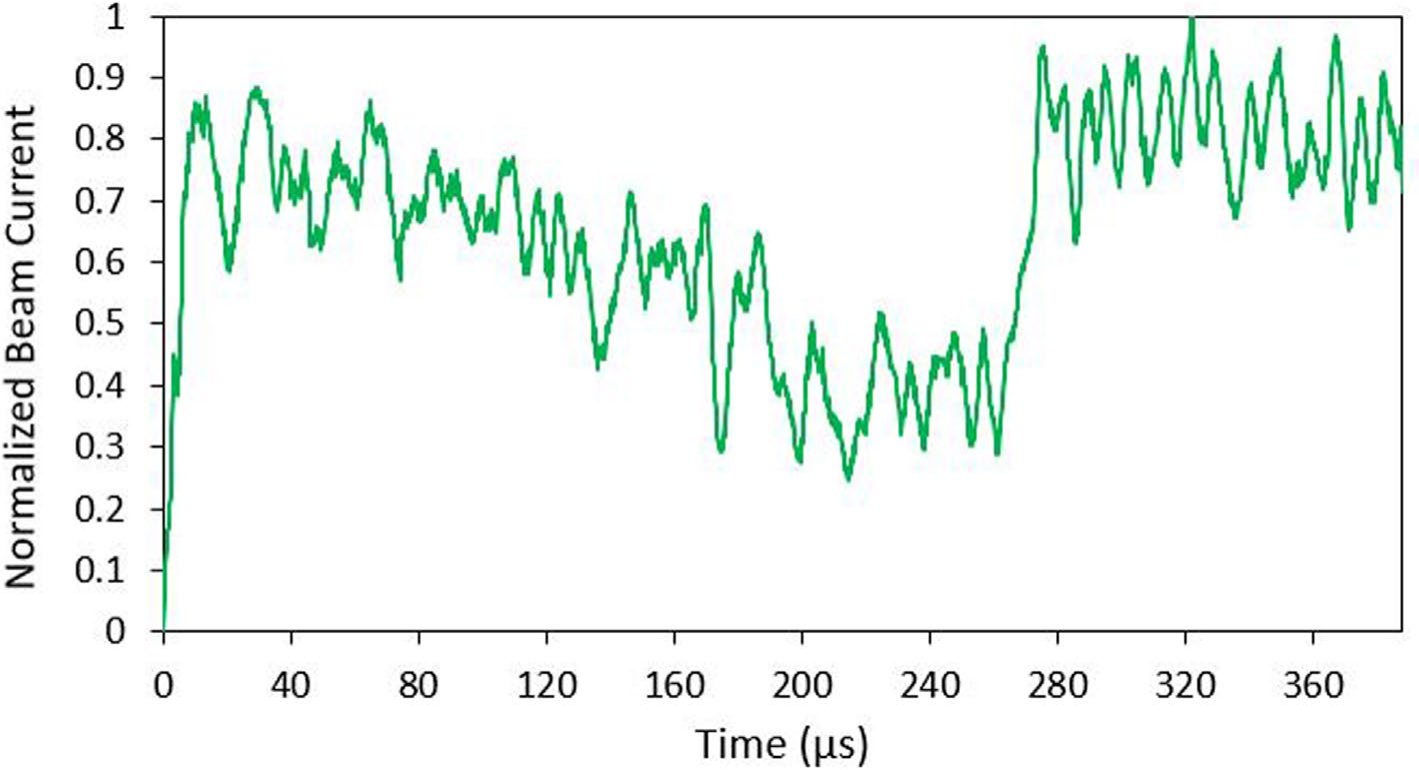
Normalized ToF data for the droplet mode emitter. The start of the plot (time = 0) is when the BNG is deactivated. A current jump around 300 $\mu$s indicates the presence of droplets

Droplet mode ToF measurements and filtering were performed in the same process as the ion mode emitter. The total current measured at the collector during ToF testing was on the order of 250 nA as seen in Fig. 21. ToF data indicated ion mode current measurement being three times larger than the droplet mode emission. The droplet mode emitter operated in the mixed ion-droplet regime meaning that both ions and liquid droplets were emitted. ToF results show multiple species of ions in the first 10 $\mu$s. This is followed by a step current increase at  275 $\mu$s indicating the presence of larger charged liquid droplets. To better present the ToF data, the current was normalized in Fig. 22 starting from the point where the BNG was deactivated (time = 0). In order to show both ion and droplets, the normalized data was presented over 400 $\mu$s.

Based on the timings of the current step increases, the positive current beam composition consisted of around 44$\%$ monomer ions, 39$\%$ dimer ions, and 17$\%$ droplets. The ToF data was used to indirectly calculate the emitter’s mass flow rate, thrust, and specific impulse. Thrust was calculated to be approximately 14.5 $\mu$N, and specific impulse of 140.6 s. A summary of the measure droplet mode parameters is presented in Table 3. The noise seen in the ToF results are most likely a result current amplifier limitations. Most of the testing used the amplifier at its most sensitive settings in order to capture changes in the nN range. Indeed, similar unfiltered noise is also observed in literature TOF studies using the same amplifier [48].

**Table 3:** Droplet mode emitter performance results summary. Droplet mass was calculated using ToF measurement. Uncertainty based on data variance not including systematic error. Thrust and specific impulse are presented as high-end approximation

	Atomic Mass (*amu*)	Expected Flight Time ($\mu s$)	Measured Flight Time ($\mu s$)	Beam Composition ($\%$)	Mass Flow Rate ($^{{\mu }g/}\!\!/_{s}$)	Upper limit of Thrust ($\mu N$)	Upper limit of Specific Impulse (s)
EMI$^+$	111	5.47	3.4 ±0.6	44 ±6	0.0103		
(EMI-BF$_4$)EMI$^+$	309	9.12	9.4 ± 1.2	39 ±6	0.025	14.5	140.6
(EMI-BF$_4$)$_2$EMI$^+$	507	N/A	N/A	N/A	N/A		
Droplets	281,003	N/A	275 ± 2	17 ± 10	10.5		

### Ion mode discussion

Full beam testing of the ion mode emitter had a measured current range of ±2 $\mu {A}$. This was close to the ESPET predicted value (3.68 $\mu$A). The indirectly calculated thrust (0.14 $\mu$N) was very close to the value predicted by ESPET (0.33 $\mu$N). Interestingly, the calculated specific impulse (4040 s) was more than double the value predicted by ESPET (1865 s). This can be attributed, in part, to the lower mass flow rate and that ESPET predicted the ion emitter would operate in the mixed ion-droplet regime instead of the experimentally demonstrated PIR. This lower flow rate can be attributed to the inefficiency of the porous wedge emitters. ESPET predicts a consistent number of emission sites based on the length of the emitter. A recent study of porous wedge emitters has shown that emission sites are not necessarily evenly distributed. During operation, the number of sites can be unpredictable and dependant on the applied voltage [34]. Although this was not experimentally verified, it is likely that the ion mode wedge emitter had fewer than expected emission sites, leading to a lower total mass flow rate and thrust.

Results indicated that the ion mode emitter operated in the expected PIR (or near PIR). The beam composition of the ion mode was determined to consist of 42$\%$ monomers and 58$\%$ dimers with no droplets identified. Uncertainty measurements in the results were derived from the variation in values over multiple tests. The indirectly calculated values of mass flow rate, thrust, and specific impulse for each emitter are estimates of performance not exact values. The results are in line with previous ToF testing using purely ionic electrosprays with EMI-BF$_4$ propellant [37, 49, 50]. While the result indicates the PIR, there is a possibility that the emitter was operating in the mixed ion-droplet regime comprised of predominated ions. During testing the BNG was only capable of deflecting around 70$\%$ of positive current emission. The remaining 30$\%$ which was not deflected could potentially include larger ion species and liquid droplets. As identified earlier, the calculated performance parameters should be seen as high-end approximation of the collected data.

The Carbon Xerogel samples produced in-house performed as expected as the ion mode emitter substrate. The targeted average pore size for the substrate was in the range of 500-700 nanometers. The final batch used for the ion mode emitters had an average measured pore size of 1.204 microns as seen in Fig. 8. Even though this result was double the anticipated pore size, this batch was used in emitter fabrication, since the pore size was considered sufficient to achieve purely ionic emission. The laser ablation of the ion mode emitters worked well, forming the desired wedge emitter with an emitter apex radius of curvature of 10 microns. It should be noted that the emitters were quite delicate and should be carefully handled as two of the three fabricated emitters were destroyed by minor abrasions during thruster assembly. When the Xerogel samples went through the drying process, the edges of the samples became warped. The warped surfaces made it challenging to produce surfaces which were perfectly flat and level. Sanding the substrates to produce a flat surface after the emitters had been laser ablated was a challenging process and strategies should be improved in the future.

### Droplet mode discussion

The full beam current measurements of the droplet mode emitter (20 - 80 $\mu$A) were a magnitude lower than the values predicted using ESPET (275 $\mu$A). From the ToF testing, the measured thrust (14.5 $\mu$N) and mass flow rate (10.5 $^{{\mu }\textrm{g}}/_{\!\!/\textrm{s}}$) were also a magnitude lower than the expected values (543 $\mu$N, 448 $^{{\mu }\textrm{g}}/_{\!\!/\textrm{s}}$). The measured specific impulse of 140.6 s which was close to the expected value, demonstrates the shortcomings of the droplet mode emitter. Other forms of propulsion such as cold-gas thrusters [51] and resistojets [52] reliably produce thrust in the mN range with a comparable specific impulse to the droplet mode emitter. In its current state the droplet mode emitter could be considered a low thrust, and low efficiency form of propulsion. Two potential reasons for measured thrust being lower than expected are measurements being taken in the stable emission regime and the lack of uniformity of the droplet substrate emitter tip.

Even with lower-than-expected performance, the droplet mode emitter is part of an integrated multimodal system, using the same propellant as the ion mode emitter. This makes the droplet mode emitter an integral part of a more versatile system, instead of a standalone form of propulsion. If the expected thrust levels were achieved, it would further improve the system’s viability.

During full beam testing the droplet mode emitter operated in either a stable low current regime or an unstable high current regime. Time-of-flight (ToF) measurements were taken during the more stable, low current regime. The total current measured at the collector during ToF testing was lower than expected in the range of 100s of nA. These lower beam current readings can be attributed to data points being collected during the consistent emission regime identified in the full beam measurements, and not during the high current regime. It was expected that the droplet mode emitter would either emit purely droplets or primarily droplets. The ToF results indicated an emission comprised of around 83$\%$ ions and 17$\%$ droplets. If the ToF measurements had been taken in the high current regime, it is likely that the beam composition would be more heavily weighted with droplets. The dramatic increase of current and prevalence of short circuits occurring indicate that emission with a higher proportion of droplets was occurring. If this was the case, then the actual thrust being produced during the high current regime may have been closer to the expected range. It is interesting to note that producing uniform porous wedges from the P3 borosilicate glass was more challenging compared to the Carbon Xerogel. The glass was chosen because its larger pore sizes would allow for an increased propellant flow rate. These larger pores also made it difficult to produce a uniform emitter edge radius of curvature to the designed 10 $\mu$m. This lack of uniformity likely lead to fewer than expected emission sites along the edge of the emitter. This can be somewhat confirmed by post-test imaging, which showed brown EMI-BF$_4$ residue on small portions of the emitter’s surface. An additional reason for the variance between the theoretical and experimental results could be the limitations of the experimental set up. The full beam and ToF setups were used independently. This implied that a single test could not collect both the full beam data and ToF measurements at the same time. As expected, in order to switch between full beam and ToF tests, high vacuum was cleared and several components had to be changed before re-pressurizing the vacuum chamber. It should be noted that the presented results for both the ion and droplet emitter show a good example of longer duration test. Some tests ended prematurely due to electrical short circuiting but these observations are consistent with observations in the literature after dozens of tests with the same emitter: chemical degradation and contamination lead to later experimentation with the same prototype to having inferior results. The physical challenges of electrospray propulsion in well documented in literature and in future work, emission characteristics and stability for both modes will be further explored as well as methods of increasing stability over the course of experiments, namely by in-house manufacturing of higher quality emitters which would require lower starting voltages [53]. Neutralization of the ion mode emitters is achieved by using bipolar operation. In our case, bipolar operation with the droplet mode emitter would potentially be impacted by charge concentration within the propellant. Future testing will investigate neutralization impacts to the droplet emitters emission and longevity implications.

## Concluding remarks

The potential utility of a multimodal electrospray thruster to provide propulsion for small spacecraft has been investigated. The novel thruster incorporated an electrospray emitter made highly-dense porous material (Xerogel) producing a highly efficient (but low thrust) mode of operation, while a second emitter made of a lower-density porous material (glass) was operated in a mixed ion-droplet regime producing a higher thrust (but lower efficiency) mode. The ion mode emitter operated in the purely ionic regime producing an estimated thrust of 0.14 $\mu$N and specific impulse of 4040 s. The glass emitter operated in the mixed ion/droplet regime producing an estimated thrust of 14.5 $\mu N$ and a specific impulse of 140.6 s. Both emitters used the propellant EMI-BF$_4$ from a common reservoir. The two emitters have demonstrated that both a high efficiency and high thrust porous electrospray can be formed into a multimodal system. The measured thrust was lower than expected for both emitters and could be attributed to an inconsistent number of emission sites, the roughness of the droplet emitter apex, and the variance between full beam and ToF testing. Future work will refine the proof-of-concept and scale up with larger emitter arrays, to demonstrate that the concept can operate as an effective multipurpose propulsion system for satellites operating in low earth orbit.

## Data Availability

No datasets were generated or analysed during the current study.
